# QuickFit: A High-Throughput RT-qPCR-Based Assay to Quantify Viral Growth and Fitness In Vitro

**DOI:** 10.3390/v16081320

**Published:** 2024-08-19

**Authors:** Nicolas M. S. Galvez, Maegan L. Sheehan, Allen Z. Lin, Yi Cao, Evan C. Lam, Abigail M. Jackson, Alejandro B. Balazs

**Affiliations:** Ragon Institute of Massachusetts General Hospital, Massachusetts Institute of Technology, Harvard University, Cambridge, MA 02139, USA

**Keywords:** viral growth, viral fitness, high-throughput, HIV, p24 ELISA, RT-qPCR, QuickFit

## Abstract

Quantifying viral growth rates is key to understanding evolutionary dynamics and the potential for mutants to escape antiviral drugs. Defining evolutionary escape paths and their impact on viral fitness allows for the development of drugs that are resistant to escape. In the case of HIV, combination antiretroviral therapy can successfully prevent or treat infection, but it relies on strict adherence to prevent escape. Here, we present a method termed QuickFit that enables the quantification of viral fitness by employing large numbers of parallel viral cultures to measure growth rates accurately. QuickFit consistently recapitulated HIV growth measurements obtained by traditional approaches, but with significantly higher throughput and lower rates of error. This method represents a promising tool for rapid and consistent evaluation of viral fitness.

## 1. Introduction

Understanding viral fitness and how treatments apply selection pressure on viral populations is pivotal for developing drugs that prevent or treat infectious diseases [1]. Viral fitness can be assessed at either the level of individual host infectivity or viral spread at the population level [2]. Changes in the fitness of many viruses causing diseases in humans, such as HIV, influenza, and SARS-CoV-2, have been extensively reported using *in vitro* and *in vivo* models to study drug resistance and immunological escape [1,2,3]. Most *in vitro* approaches consider the outgrowth of viruses in cell cultures, followed by the measurement of either genetic material or viral antigens. Assays based on quantifying the viral genetic material are typically more reliable than those quantifying viral proteins but are more expensive to run [4,5,6]. However, the intrinsic measurement error in current technologies, coupled with the stochasticity of viral infection and replication, results in significant uncertainty in the existing measurements of viral growth.

Despite notorious public health initiatives and research efforts to develop therapies and vaccines, HIV is still a significant global pandemic [7]. Highly active antiretroviral therapy (HAART) has been used for the past three decades to treat or prevent HIV infection [8,9,10], and several drug classes have been developed targeting key steps in the viral life cycle, including reverse transcription (RT), integration, and viral particle maturation [11,12,13]. Most regimens combine two or three RT inhibitors, and strict adherence to the prescribed drug regimen is essential as the viral load rebounds within weeks of treatment interruption [10]. As such, the development of long-lasting and curative strategies for HIV remains a priority for the field [14].

It has been extensively reported that suboptimal concentrations of early generations of HAART lead to the natural emergence of mutations in the RT polymerase, rendering the virus resistant to drugs [15,16,17]. However, these resistance-associated mutations usually incur a cost in terms of the viral replication rate, a phenomenon usually referred to as fitness cost [18,19,20]. Accurately determining the viral fitness of escape mutants is crucial to the development of combinations of drugs that can effectively apply selection pressure on the virus, as mutations incurring higher fitness costs are less likely to arise [20]. Existing approaches to determining the viral growth and fitness of HIV and other viruses rely on either a culture of the virus in cells *in vitro* or animals [21]. Longitudinal samples are subjected to either ELISA- or RT-qPCR-based methods to measure viral antigens or genetic material [22]. However, these approaches are time-consuming, expensive, and have varying degrees of sensitivity [23,24], which leads to inconsistent reports of growth rates in the literature [25,26,27].

To address these limitations, we developed a high throughput, low-cost pipeline that could systematically be used to study viral fitness across different research centers. We developed an approach that is agnostic to differences in viral titers between stocks and took advantage of improvements in high-throughput liquid handling to minimize the hands-on time and maximize useful data. We employed RT-qPCR, given its wide dynamic range and high accessibility, to conduct an assay we termed QuickFit. To validate the method, we evaluated the relative growth rate of different HIV strains and compared them to those generated by a widely used p24-based ELISA. For each HIV strain tested, QuickFit generated robust and reproducible estimates of the viral growth with higher precision than ELISA. To evaluate the fitness cost of drug escape mutations within the HIV RT *polymerase* gene, we measured the growth rates of viruses harboring known RT mutations. QuickFit was also used to determine the fitness cost imposed on viruses grown in the presence of emtricitabine and the doubling time of numerous HIV isolates, finding significant differences in their growth rates.

## 2. Materials and Methods

**Isolation and Activation of CD4^+^ T cells**

Primary human PBMCs were isolated from human blood samples by Ficoll density gradient centrifugation, as reported previously [28]. Briefly, blood was diluted in a ratio of 1:2 in 1x DPBS (Corning, NY, USA, #21031CV) and gently layered over a Ficoll buffer (Cytiva, Marlborough, MA, USA, #17144002) in a 50 mL conical tube. Diluted blood was then spun at 400× *g* for 40 min. The resulting monolayer of cells was isolated and washed using RPMI1640 (StemCell Technologies, Cambridge, MA, USA, #36750) supplemented with 10% FBS (VWR, Radnor, PA, USA, #97068-085), Pen/Strep (Thermo, Waltham, CA, USA, #15140122), L-Glutamax (Life Technologies, Carlsbad, CA, USA, #25030081), MEM non-essential amino acids (Corning, #25-025-CI), HEPES (Thermo, #MT25060CI), and sodium pyruvate (Corning, #25-000-CI) (isolation media). PBMCs were counted and frozen in 90% FBS and 10% DMSO at a density of 20 × 10^6^ cells per tube. Then, CD4^+^ T cells were isolated from frozen PBMC tubes using the EasySep™ Human CD4^+^ T Cell Isolation Kit (StemCell Technologies, #17952) following the manufacturer’s protocol. Isolated naive CD4^+^ T cells were resuspended in isolation media supplemented with recombinant IL-2 (R&D Systems, Minneapolis, MI, USA, #202-IL-050) at 10 ng/mL (growth media) and 4 µg/mL of an anti-CD28 antibody (Biolegend, San Diego, CA, USA, #302934). Cells were plated in non-TC-treated 24-well plates previously coated with 2 µg/mL of an anti-CD3 antibody (Biolegend, #317304) overnight at 4 °C. Cells were incubated at 37 °C and 5% CO_2_ for 2 to 4 days until proliferation was visible. Cells were then washed with isolation media and pooled with growth media in a T75 flask for another 2–4 days before use in downstream assays. The purification and activation efficiency were routinely assessed by flow cytometry. All the experiments presented here were performed using PBMCs from the same donor to reduce variability unless stated otherwise.

**Infection of Primary Human CD4^+^ T cells with HIV-1 Infectious Molecular Clones**

HIV-1 viral stocks were obtained by transfecting 293T cells (ATCC, Manassas, VA, USA, #CRL-3216) with full-length replication-competent infectious molecular clone (IMC) plasmids, as described previously (ATCC, BEI Resources, #ARP114, #ARP2708, ARP#3552, #ARP11746, #ARP13402) [29]. All IMC plasmids (both WT and mutants) were routinely full-length sequenced to confirm their accuracy. The TCID_50_ and infectivity of viral stocks were routinely assessed on TZM-bl cells (ATCC, BEI resources, #ARP8129) prior to any assays, as previously described [30,31,32,33,34]. HIV-1 viral stocks were then diluted in duplicate in growth media in 96-well flat bottom plates. Activated CD4^+^ T cells were added to the plate at a density of 1 × 10^5^ cells/well. Plates were then spinoculated at 400× *g* for 1 h and incubated overnight at 37 °C and 5% CO_2_. After 12 h, the cells were transferred to 96-well round bottom plates and washed 3 times with isolation media to remove unbound viral particles by spinning them down at 500× *g* for 5 min and removing the media. After washing, the cells were resuspended in 200 µL of growth media and incubated at 37 °C and 5% CO_2_ for 6 days. If the samples were treated with emtricitabine (Emtriva^TM^ FTC, Gilead Sciences, Foster City, CA, USA, #61958-0601), growth media was supplemented with 70 ng/mL in all steps. As a vehicle control, the same volume of DMSO was added to the growth media of the cells that were not treated with FTC.

**Supernatant collection and RNA extraction**

Viral RNA extraction was performed using the one-step 5 min protocol reported recently [35,36,37]. Viral supernatants were collected starting 48 h after the cells were transferred to round bottom 96-well plates until the end of the assay. A total of 24 µL of supernatant was collected daily in a 384-well PCR Plate (Santa Cruz Biotechnology, Dallas, TX, USA, #sc-205893) by a TECAN Fluent 780 Liquid Handler and fresh media with the same composition as the one collected was added back to the culture plates to recover the original volume. Upon collection, 8 µL of QuickExtract DNA Extraction Solution (LGC Biosearch Technologies, Teddington, USA, #QE09050) were immediately added to the supernatant, vortexed for 5 s, spun for 5 s at 1000× *g*, and then incubated in a thermocycler at 95 °C for 5 min and 4 °C for 5 min to finish the extraction [35,36,37]. The extracted viral RNA was then stored at −80 °C until the RT-qPCR was performed. QuickExtract DNA Extraction Solution freeze–thaw cycles were limited to no more than 3 to prevent decreased effectiveness. Alternatively, supernatants were immediately frozen so protein lysis could be performed later.

**p24 ELISA quantification**

p24 capsid concentration in viral supernatants was quantified with the Leidos Biomedical Research HIV-1 p24CA Antigen Capture Assay Kit, following the manufacturer’s instructions. Briefly, the 96-well ELISA plates (Thermo #446612) were coated with a 1:400 dilution of the capture antibody (Lot#PP292-3) in DPBS and incubated overnight at 4 °C. Plates were then blocked for one hour and washed six times with a wash buffer. During this incubation, frozen samples were thawed and protein lysis was performed by adding 1 µL of 10% Triton X-100 (Thermo #BP151-500) in dH_2_O to 19 µL of sample, then incubating this for 1 h at 37 °C. Samples were then diluted in sample diluent, added to the plates, and incubated for 2 h at 37 °C. All samples were run in duplicate. Independent standard curves diluted in sample diluent were held for each plate (Lot#SP968T). The plates were washed as before, then a 1:300 dilution of the primary antibody (Lot#SP2143A) in the corresponding diluent was added to the plates. Plates were incubated for 1 h at 37 °C, washed six times again, and a 1:25,000 dilution of the secondary antibody (Goat anti-Rabbit IgG H+L Chain HRP Conjugated—Bethyl #A120-201P) in the corresponding diluent was added to the plates. The optimal secondary antibody concentration was previously determined by testing several dilutions of this reagent with dilutions of the standard curve. After incubating for 1 h at 37 °C, the plates were washed one last time as before and once with DPBS. For the readouts, 100 µL of TMB substrate (VWR #95059-156) was added to the wells and incubated for 30 min at room temperature. The reactions were stopped by adding 100 µL of 1N HCL, and the OD_450_ and OD_650_ values were read within five minutes in an Emax Laboratory Precision Microplate Reader. The OD_650_ was subtracted from the OD_450_, and the p24 concentrations were determined by interpolating these values into each independent standard curve using a four-parameter equation.

**Quantification of viral gene copies by RT-qPCR**

The extracted viral RNA was thawed, and 5 µL were used in a 10 µL RT-qPCR reaction with the qScript XLT one-step RT-qPCR Tough Mix, low ROX kit (Quanta Biosciences, #95134-500), a TaqMan probe (56-FAM/CCCACCAAC/ZEN/AGGCGGCCTTAACTG/3IABkFQ), and primers designed to target the Pol gene of HIV_REJO.c_, (5′-CAATGGCCCCAATTTCATCA and 3′-GAATGCCGAATTCCTGCTTGA), HIV_NL4-3_/HIV_JR-CSF_/HIV_89.6_ (5′-CAATGGCAGCAATTTCACCA and 3′-GAATGCCAAATTCCTGCTTGA), and HIV_BF520_ (5′-CAATGGCAGCAATTTCACCA and 3′-GAATCCCAAATTCCTGTTGGA) (Integrated DNA Technologies, IDT). A different TaqMan probe was used to detect HIV_BF520_ (56-FAM/CCCACCAAC/ZEN/AGGCTGCTTTAACTG/ZEN/3IABkFQ/). The samples were run on a QuantStudio 12K Flex (Thermo, Applied Biosystems). The following cycling conditions were used: 50 °C for 10 min, 95 °C for 3 min, followed by 55 cycles at 95 °C for 3 s and 60 °C for 30 s. Viral loads (Genome copies (GC)/mL) were determined by interpolating the CT values into a standard curve generated with RNA extracted from a previously titered serially diluted viral stock. The range of the assay was 1 × 10^4^ to 1 × 10^9^ GC/mL.

**Growth rate quantitation and growth curve modeling**

To quantify the growth rates and model growth curves, the viral growth indicator data (GC/mL or p24 pg/mL) were either filtered or not filtered with the following set of rules. For the p24 ELISA data, any individual wells not reaching a value above the limit of detection on day 2, those reaching the upper limit of detection at any time, and those where p24 concentration decreased up to 10% relative to the previous quantitation were excluded; for the RT-qPCR data, any individual wells reaching the lower or upper limit of detection at any time point, those where viral loads decreased up to 10% relative to the previous quantitation, and those where the initial viral load was more than 0.25 times or less than 0.025 times the highest quantified value for that well were excluded. Viral growth indicator values were corrected to account for the corresponding dilution factor for each day after sample collection.

After filtering, viral growth indicator data were analyzed using the non-linear mixed-effect (NLME) modeling software MonolixSuite 2021R (Lixoft SAS, Antony, France) [38,39]. The following equation was used to estimate growth rate (*r*) and carrying capacity (*K*) population parameters using a continuous observation model type.

(1) $$\frac{d}{dt}V=\frac{r\times V\times K}{\left(K+V\right)}$$

**Equation (1)**: Half-maximal formula used to quantify growth rates, with *V* being the quantified viral growth indicator data and *t* being the time. For the estimation of the *r* values, random effects were allowed for each individual model. The estimation of the individual *K* values was constrained so that they would remain constant for each HIV isolate unless stated otherwise. The output viral growth indicator data obtained were used to model growth curves as they were, or by normalizing them to the initial values obtained for each individual sample. Data were then plotted using GraphPad Prism v10.2.2 (LLC).

**Ethics statement**

The use of human blood samples was approved by the Partners Institutional Review Board and verbal consent was obtained when collecting the samples.

## 3. Results

### 3.1. Parallel Viral Cultures Enable Accurate Modeling of Growth Rates

**Figure 1:**
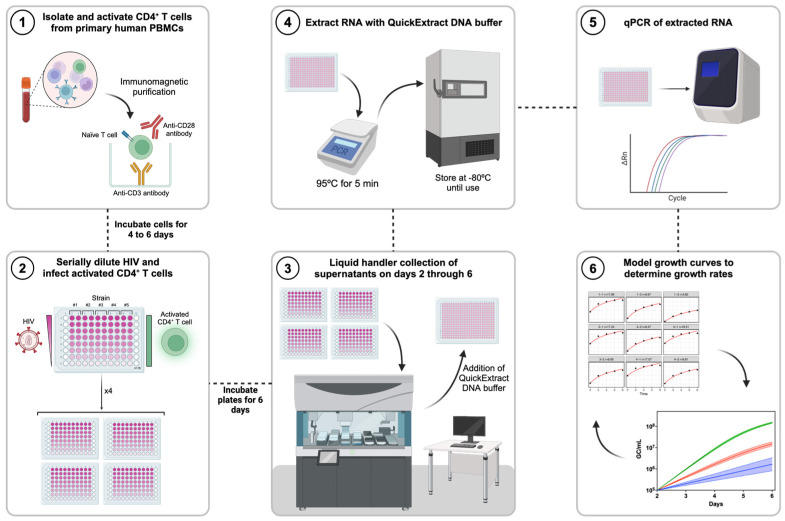
QuickFit is a high-throughput RT-qPCR-based HIV *in vitro* replication assay platform. Schematic representation of the experimental setup followed to perform QuickFit RT-qPCR evaluations of HIV growth rates *in vitro*.

CD4^+^ T cells were isolated from peripheral blood mononuclear cells (PBMCs) using immunomagnetic purification and activated via CD3/CD28 co-stimulation to maximize their susceptibility to viral infection. To reduce experimental noise, we employed 16 parallel cultures for each individual virus under evaluation. Cells were mixed with serial dilutions of each virus and cultured over a period of 6 days to allow for multiple rounds of infection and viral replication. At daily intervals, beginning 2 days post-infection, the virus-containing supernatant was collected from each individual well simultaneously using a liquid handler, immediately subjected to RNA extraction using a commercially available QuickExtract buffer, and stored at −80 °C in a 384-well plate format. After six days of growth, we determined the viral loads for each sample by performing high-throughput RT-qPCR with strain-specific primers. Viral growth rates were determined by non-linear mixed-effect (NLME) modeling simulations in the MonolixSuite software using a half-maximal equation (Figure 1).

**Figure 2:**
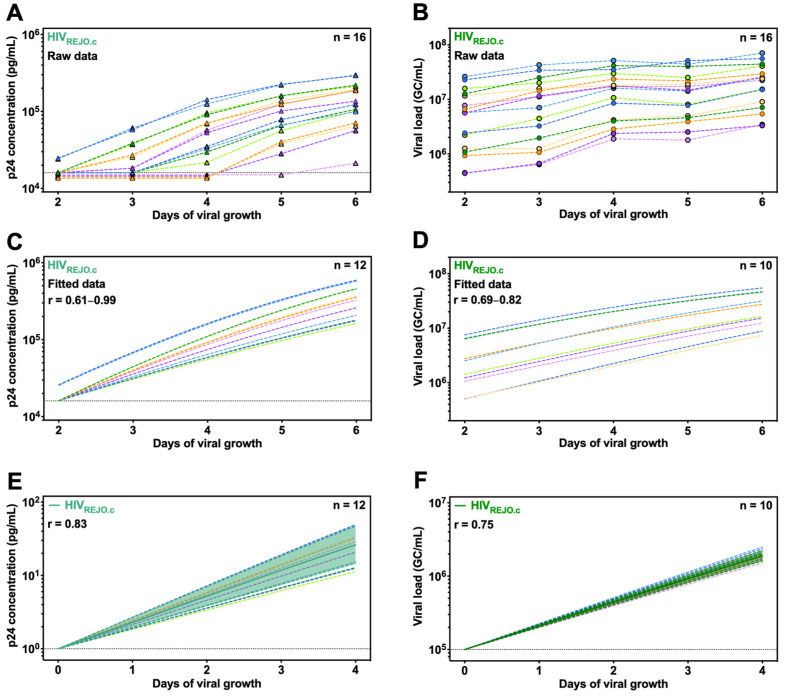
Analysis and modeling of HIV *in vitro* replication growth curves as measured by p24 ELISA and QuickFit RT-qPCR. Both p24 ELISA (left) and QuickFit RT-qPCR (right) were performed with samples obtained from HIV_REJO.c_-infected CD4^+^ T cells. (**A**) Determined raw p24 concentrations (pg/mL) and (**B**) viral loads (GC/mL) are shown. A total of 16 samples at five different time points were evaluated, and matching light–dark colors represent experimental duplicates. (**C**,**D**) Samples were subjected to inclusion and exclusion criteria, and the filtered data were used to determine growth rates and carrying capacities by running an NMLE modeling with a half-maximal equation in the MonolixSuite 2021R software. (**E**,**F**) Viral growth (p24 concentration or GC/mL) was calculated by normalizing the initial value on day 0 to 1.00 pg/mL or 1 × 10^5^ GC/mL and subsequently interpolating the remaining values from days 1 to 4 based on the fit curve model. Overlaid is the mean growth rate and the 95% confidence interval. The r value represents the average growth rate determined for all included samples by each assay. The dotted lines represent the limit of detection for each assay.

To assess the robustness of QuickFit relative to the existing p24 ELISA-based methods, we infected activated CD4^+^ T cells with serial dilutions of HIV_REJO.c_, a clade B, R5-tropic, transmitted/founder (T/F) isolate that is widely used both *in vitro* and *in vivo* [40,41]. Culture supernatants were collected daily and split into two samples that were either lysed to solubilize the p24 protein or RNA extracted to quantify the virus via p24 ELISA or RT-qPCR, respectively. Of the 16 samples evaluated via ELISA, only samples from cultures receiving the highest inoculum were above the limit of detection for the initial time point (Figure 2A). By day 4, the samples with the most and second-to-most diluted virus inoculum remained below the limit of detection (Table S1). In contrast, the samples obtained in parallel that were quantified via RT-qPCR were within the limit of detection for the assay, irrespective of the time point evaluated (Figure 2B).

We first analyzed the p24 ELISA data using an NLME model but found that including the concentrations from all wells yielded inaccurate growth estimates, as many of these were below the limit of detection at the early time points. To address this limitation, we defined the inclusion and exclusion criteria to enable consistent data analysis across the independent *in vitro* experiments. We included all the individual wells that were above the limit of detection after four days of viral growth but excluded wells that reached the upper limit of detection at any time point. In addition, we excluded wells whose signals declined over 10% between any of the time points to remove wells exhibiting inconsistent growth. Together, these filters resulted in the exclusion of four individual wells (Table S1). NLME modeling of the 12 remaining wells via the ELISA data generated growth rates (r) ranging from 0.61 to 0.99 (Figure 2C).

In contrast to the ELISA measurements, the RT-qPCR-based viral loads obtained from the RNA-extracted samples were all within the limit of detection at all time points. When analyzing the viral load data from all wells, NLME modeling generated r values ranging from 0.62 to 1.06 (Figure S1A and Table S2). To increase the reliability of the analysis, we excluded wells that reached the upper or lower limit of detection at any time point and wells in which the viral load declined by more than 10% between consecutive time points. To avoid measurements close to the carrying capacity of the assay, we excluded wells in which the viral load at the first time point was within four-fold of the highest viral load observed. To avoid measurements exhibiting growth outside of the linear range, we also excluded wells in which the viral load at the last time point was more than 40-fold higher than the initial time point. This resulted in the exclusion of six individual wells (Table S1), and NLME modeling of the remaining wells yielded r values ranging from 0.69 to 0.82 (Figure 2D). Both p24 and qPCR-based NLME modeling were fitted to a shared carrying capacity to enable direct comparisons between the two assays.

To visualize the average r value and error in these measurements, we simulated viral growth in silico starting from either a 1 pg/mL p24 concentration or a 1 × 10^5^ GC/mL viral load using the previously determined growth rates. The average r value for the 12 individual wells analyzed with ELISA was 0.83, with a standard deviation of 0.12 (Figure 2E and Table S2). For the qPCR data, the average r value across all 16 wells was 0.80, with a standard deviation of 0.12 (Figure S1B,C and Table S2), whereas after excluding the unreliable wells, the average r value across the ten remaining wells was 0.75, with a standard deviation of 0.04 (Figure 2F and Figure S1D, and Table S2). Overall, the growth rates determined for HIV_REJO.c_ by QuickFit were similar to those obtained by ELISA but with significantly less variability and substantially less hands-on time, highlighting the advantages of QuickFit over conventional methods of evaluating viral growth.

### 3.2. Comparison of HIV_REJO.c_ Mutant Growth Rates via QuickFit

After validating QuickFit using a wildtype virus, we sought to measure differences in the fitness costs of individual mutations within HIV_REJO.c_ polymerase. Mutations M184I and M184V, which spontaneously emerge in people living with HIV who receive early generations of HAART, lead to drug resistance at the expense of fitness, by reducing reverse transcriptase processivity [42,43]. Prior studies have described a range of impacts on viral replication kinetics and titers when comparing the WT and Pol mutant strains [43,44]. We introduced the Pol M184I and M184V mutations into the HIV_REJO.c_ infectious molecular clone (IMC) and prepared working viral stocks. We infected activated CD4^+^ T cells with WT or Pol mutant strains and collected supernatants to perform p24 ELISA and RT-qPCR. As these mutants have been shown to confer resistance to the antiretroviral drug emtricitabine (FTC), we also included wells treated with 70 ng/mL (300 nM) of FTC, the equivalent of 0.5× times the EC_50_ value reported for this drug *in vitro* [45]. Control samples were given the same volume of DMSO diluent without the FTC.

**Figure 3:**
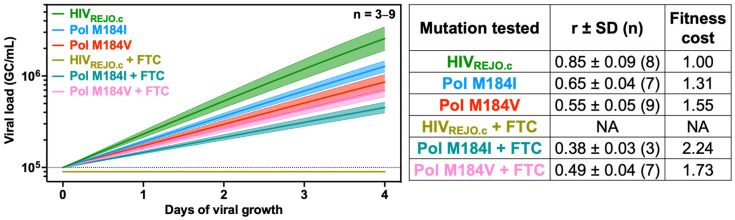
Comparative assessment of replication rates among HIV_REJO.c_ mutants for the Pol gene in the presence of antiretroviral drugs. Single-point mutants for the Pol gene were generated using the HIV_REJO.c_ IMC backbone. Then, QuickFit RT-qPCR was performed and growth rates were determined. To evaluate the impact of ART drugs in these mutants, these strains were also grown in the presence of 300nM of emtricitabine (FTC, 0.5× the EC_50_ *in vitro* value) or DMSO as a control. Viral growth (GC/mL) was calculated by normalizing the initial value of each sample (after inclusion and exclusion criteria filtering) on day 0 to 1 × 10^5^ GC/mL and by subsequently interpolating the remaining values from days 1 to 4 based on the fit curve model. Overlaid is the mean growth rate and the 95% confidence interval. The table to the right shows the r values (growth rates) ± the standard deviation for each strain tested for both conditions. The sample size (n) references the number of wells included in the analysis after filtering with inclusion and exclusion criteria. The fitness cost represents the doubling rate of each strain relative to the WT HIV_REJO.c_ without FTC. The dotted line represents the limit of detection for each assay. NA: No growth was detected.

Since these mutants shared the same parental strain, we performed NLME modeling using a shared carrying capacity. The average RT-qPCR r value generated for the WT HIV_REJO.c_ was 0.85 (±0.09) (Figure 3). As previously reported, both Pol mutations resulted in decreases in the r value, which translated into slower-replicating viruses, even in the absence of drugs. To quantify these differences, we normalized the r values to that of the WT strain to determine a fitness cost metric. Relative to the WT strain, the M184I strain had a fitness cost value of 1.31 (i.e., a 31% slower replication rate), whereas the M184V strain had a fitness cost value of 1.55 (Figure 3 and Table S3).

In contrast to the DMSO-treated controls (Figure S2A,B), treatment with FTC resulted in no growth for HIV_REJO.c_ WT, as measured by both p24 ELISA and RT-qPCR (Figure S2C,D, top). However, both Pol mutants were able to grow in the presence of FTC to varying degrees (Figure S2C,D, middle and bottom). Since HIV_REJO.c_ WT did not grow in the presence of the drug, the fitness cost values for M184I and M184V in the presence of FTC were normalized to the WT strain grown without the drug. For M184I, this fitness cost was 2.24, whereas for M184V the fitness cost was 1.73. Therefore, although these mutant strains are still able to grow in the presence of ART, they grow better in the absence of the drug. We found similar r values using data generated by ELISA, but with significantly greater variability which masked differences between each condition evaluated (Figure S2E and Table S3).

Sources of variability when evaluating HIV growth and fitness include both intrinsic donor PBMC differences as well as the degree of cellular activation between experiments. To address the robustness of QuickFit across donors, we infected activated CD4^+^ T cells from three different individuals in three independent experiments with HIV_REJO.c_ and the Pol M184I strain (Figure S3). No statistical differences were seen between any of the three donors tested when comparing the growth rates for HIV_REJO.c_ WT or the Pol M184I strain (Figure S3A). Accordingly, growth curves for each strain were within the measurement error for all donors (Figure S3B), and pairwise comparisons found that the fitness of the M184I mutant relative to WT was very similar, irrespective of the donor (Figure S3C). These data suggest that donor-to-donor variability does not significantly impact QuickFit when comparing mutants of the same strain.

Finally, to confirm that the growth rates determined for the FTC-treated samples were not impacted by the presence of the RT inhibitor drug in the culture supernatants, we performed RT-qPCRs with serial dilutions of previously titered HIV_REJO.c_ viral stocks spiked with increasing concentrations of FTC (ranging from 35 ng/mL (60nM) to 700 ng/mL (30µM)). No differences were observed for any of the raw CT values across different viral dilutions or FTC concentrations (Figure S4). Accordingly, the CT values reported in the presence of FTC or DMSO were the same as those of non-spiked viral stocks. Altogether, these results demonstrate that QuickFit RT-qPCR can be used to reliably determine growth rates for mutant strains of the same HIV isolate.

### 3.3. The High Sensitivity of QuickFit Allows for the Evaluation of Different HIV Isolates

To quantify the differences in HIV growth rates between isolates, we tested four different HIV strains: HIV_NL4-3_, a clade B, X4-tropic, chronic isolate; HIV_JR-CSF_, a clade B, R5-tropic, chronic isolate; HIV_89.6_, a clade B, dual-tropic (X4- and R5-tropic), chronic isolate, and HIV_BF520_, a clade A, R5-tropic, T/F isolate (ATCC, BEI Resources, #ARP114, #ARP2708, ARP#3552, #ARP13402, respectively) [46,47]. All isolates could be detected by p24 ELISA and RT-qPCR, albeit with differences in sensitivity (Figure S5). Robust detection of p24 was observed for HIV_NL4-3_, HIV_JR-CSF_, and HIV_89.6_ (Figure S5A,C,E). However, HIV_BF520_ was difficult to detect, with only the two wells with the highest initial input of the virus reaching values above the limit of detection by day 2 (Figure S5G). In contrast, RT-qPCR-based viral loads were all above the limit of detection for HIV_NL4-3_ and HIV_89.6_, while 14 out of the 16 samples for HIV_BF520_ and HIV_JR-CSF_ were detectable at the first time point (Figure S5B,D,F,H).

**Figure 4:**
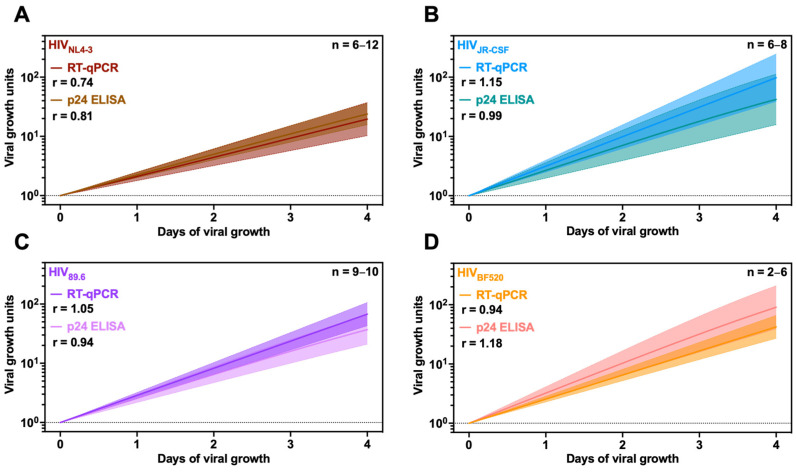
Evaluation of viral growth of four different HIV-1 isolates by p24 ELISA and QuickFit RT-qPCR. (**A**) HIV_NL4-3_, (**B**) HIV_JR-CSF_, (**C**) HIV_89.6_, and (**D**) HIV_BF520_ growth rates were determined using the QuickFit pipeline by p24 ELISA (lighter shades) and RT-qPCR (darker shades). Viral growth (p24 concentration or GC/mL) was calculated by normalizing the initial value on day 0 to either 1.00 pg/mL or 1.00 GC/mL and subsequently interpolating the remaining values (filtered by inclusion and exclusion criteria) from days 1 to 4 based on the fit curve model. Overlaid is the mean growth rate and the 95% confidence interval. The r value represents the average growth rate determined by each assay for all included samples. The dotted lines represent the limit of detection for each assay.

We modeled each virus’ growth rate and carrying capacity independently, finding that the average r values and standard deviations determined by either ELISA or RT-qPCR were similar for the four strains (Figure 4 and Table S4).

**Figure 5:**
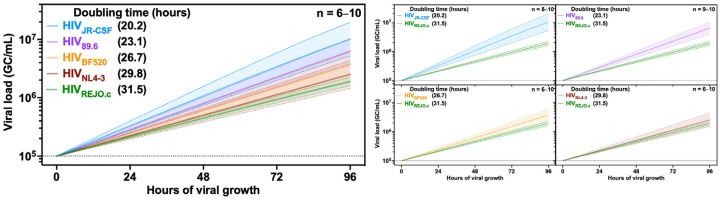
Different HIV strains exhibit different growth rates and carrying capacities. Comparison of HIV_REJO.c_, HIV_NL4-3_, HIV_JR-CSF_, HIV_89.6_, and HIV_BF520_ growth rates determined by QuickFit RT-qPCR. Viral growth (GC/mL) was calculated by normalizing the initial value on day 0 to 1 × 10^5^ GC/mL and subsequently interpolating the remaining values (filtered by inclusion and exclusion criteria) from days 1 to 4 based on the fit curve model. Overlaid is the mean growth rate and the 95% confidence interval. The doubling time represents the number of hours it took each isolate to double its viral load. The plots to the right show the pairwise comparison of each strain to HIV_REJO.c_. Dotted lines represent the limit of detection for each assay.

To enable direct comparison between strains, we performed a single simulation that included data from all strains without constraining the carrying capacity to a shared value, and the relative growth rates were expressed as doubling time. All strains had a doubling time of approximately 24 h, in line with previous reports [48] (Figure 5 and Table S5), with HIV_REJO.c_ and HIV_JR-CSF_ exhibiting the largest difference in growth rates. Finally, to determine whether the growth rates of different HIV isolates are impacted by donor-to-donor differences, PBMCs from three different donors were infected in three independent experiments with either HIV_REJO.c_ or HIV_JR-CSF_. A single simulation, including both strains and all donors, was performed without constraining the carrying capacity to a shared value. Interestingly, growth rates measured in the cells from donor 1 were significantly faster than those determined for donors 2 and 3 (Figure S6A). However, no differences were seen across donors when relative fitness was measured by normalizing growth rates to the HIV_REJO.c_ strain (Figure S6B). Taken together, our results demonstrate that QuickFit can be used to evaluate and compare the growth rates, carrying capacities, and fitness of different HIV strains with high precision.

## 4. Discussion

Early efforts to determine HIV growth and fitness were based on kinetic data obtained from culture, using either individual viruses or dual infection/growth competition experiments [49]. Individual viral growth assays rely on measuring viral proteins, genetic material, physical particles, or reporter gene expression [50]. These methods are usually time-consuming, expensive, and have different degrees of sensitivity between different viral isolates, resulting in inconsistent growth rates [25,26,27,51]. Dual infections or competitive growth assays employ a reference and a test strain to infect cells in the same culture, and the two strains are monitored via the env gene heteroduplex tracking assays, allowing quantification of their growth and fitness [49,52]. However, this fitness measuring approach is time-consuming and difficult to scale [53]. More recent iterations of viral growth assays measure reverse transcriptase activity and perform linear regression analyses to determine growth kinetics, not only for HIV but for lentiviruses in general [54,55]. Other novel approaches measuring viral growth aim to streamline this process with high-throughput by performing parallel replication capacity assays followed by infection of reporter cell lines, such as TZM-bl; however, these measurements are indirect [56]. Dual infection cultures and allelic-specific PCRs can also be used to evaluate fitness differences between strains, yet these are difficult to scale efficiently [55,57,58].

Since a significant portion of viral growth measurements rely on ELISA-based approaches, we compared QuickFit to the growth rates measured this way. ELISA assays have numerous sources of variability, including antibody concentrations, number of washes, sample dilution, and both the time and temperature of incubation for each step, leading to differences in results between plates [59,60]. In our own studies, we found significant variation in the recognition of the HIV p24 capsid protein among different strains. In particular, HIV_NL4-3_ exhibited the strongest signal, while HIV_BF520_ was only detectable at the highest inoculums, leading to wider measurement error. Protein alignment of the p24 capsid proteins of HIV_NL4-3_ and HIV_BF520_ resulted in a pairwise identity of 91.3%, emphasizing that even relatively minor differences in antigens can have large impacts on the sensitivity of p24-based ELISAs [25]. These issues can be minimized by incorporating standard curves to account for plate-to-plate variations but may still contribute to the observed growth rates [61].

We developed QuickFit as a simpler and more robust method for the quantitation and analysis of viral growth and fitness, drawing from the advantages seen in previously reported approaches. Previous methods of RNA extraction were significantly more time-consuming, requiring at least a few hours to obtain the extracted genetic material and relying on several buffers and purification columns for them to work. The incorporation of a novel RNA extraction reagent enabled the use of high-throughput liquid handling in sample processing to minimize the hands-on time [35,36,37,62]. Measuring the growth rate of a single virus using QuickFit employs 80 distinct samples, representing approximately 20% of a 384-well plate. In contrast, p24 measurements obtained via ELISA require a full 96-well plate. As an RT-qPCR-based assay, QuickFit reliably measures even relatively small changes in fitness between different HIV strains [63,64]. While we employed universal primers targeting highly conserved regions of the HIV Pol gene, optimal sensitivity of these assays could be attained by precisely matching the sequence of the primers and probe to each strain [62]. Not including the time needed to collect supernatants over multiple days, QuickFit can produce results in as little as 3 h. Remarkably, previously developed RT-qPCR-based assays required around 2 h for sample collection and 4h to perform the actual assay not including sample analysis, highlighting once again the improved speed of QuickFit [62,65,66].

To reduce the intrinsic variability in viral growth that arises from *in vitro* culture, we analyzed QuickFit data on all 80 samples obtained across 16 wells for each individual virus to derive exclusion criteria that would minimize error. We excluded wells exhibiting decreasing viral load over time, as these were interpreted as failed cultures. We also excluded wells that reached the carrying capacity of the assay before the final time point, as well as any wells that failed to reach logarithmic growth, which has been shown to occur when the initial virus inoculum is too low [67,68]. The need for these criteria to improve the reliability of QuickFit measurements is a consequence of using raw viral stocks, eliminating the need to titer each viral preparation.

Numerous studies have proposed mathematical models of HIV replication and growth determination *in vivo* and *in vitro* [49,67,68,69,70]. The parameters considered in these models include the number of viral particles, the doubling time or growth rates, the carrying capacity, and the time span of the experiment. The use of a half-maximal equation via NLME modeling enables constraining some of these variables when required [38,39,71]. In our case, when determining the growth rates for each individual well infected with the same HIV strain, we constrained the carrying capacity to a shared value [72,73]. The same rubric was applied when we analyzed different HIV mutant strains derived from the same backbone, namely mutations in polymerase. Of note, analyzing these metrics for each Pol mutant strain independently resulted in somewhat different growth rates and carrying capacities compared to those analyzed together. However, each metric was based on a limited number of wells that fell within the acceptable range of growth conditions, making them less reliable [73,74]. When comparing growth rates of different HIV strains, more accurate values were obtained by allowing the NLME model to derive separate carrying capacities for each strain. The choice to constrain these variables should be made carefully when using QuickFit to ensure accurate estimates of growth rates.

Single-point mutations within the HIV envelope or polymerase gene can have profound effects on the fitness of HIV. This directly impacts the potential for HIV to escape from antibodies or antiretroviral drugs, as each mutation introduces a unique cost or benefit to viral growth [20]. Previous reports suggest that the Pol mutant M184V has an intermediate fitness relative to the WT and the M184I strains [44,75,76]. In agreement with these findings, isoleucine at position M184 is observed more frequently in patients refractory to HAART at early time points, but valine quickly outcompetes it, becoming the dominant mutation in these patients over time [75,77,78]. In our assays, M184I grew somewhat better than M184V in the absence of FTC; however, M184V grew substantially better than M184I in the presence of the drug. This shift in fitness in the presence or absence of drugs could account for the differences in relative fitness between the mutants we observed via QuickFit compared to other reports [44].

Despite our efforts to optimize QuickFit, other sources of intrinsic error still remain, such as donor variability between the PBMCs used for each assay [79]. To address this, we evaluated the growth rates measured across three different donors and two HIV strains. Of note, the relative fitness cost within a given isolate (i.e., HIV_REJO.c_ WT and the Pol M184I mutant) remained similar, with only minor variations between donors. However, the absolute growth rates of HIV_REJO.c_ and HIV_JR-CSF_ strains measured across different PBMCs were statistically different. This could be attributed to varying levels of co-receptor expression or host genetics, which could have impacted their susceptibility to infection [79]. Despite this, by normalizing the growth rates of each donor, the relative difference between the HIV_REJO.c_ and the HIV_JR-CSF_ isolates was maintained. One potential solution to the donor-to-donor variability would be to employ cell lines instead of primary PBMCs for the HIV outgrowth assays [80,81]. However, this presents additional caveats, as each cell line would exhibit different phenotypes relative to PBMCs, such as their surface CD4, CXCR4, and CCR5 expression levels, the internalization rates of these receptors upon HIV infection, and their intracellular dNTP availability [44,82].

Despite these caveats, QuickFit represents an improved platform for determining viral growth rates and evaluating fitness costs and could be further adapted to enhance its versatility. For instance, future iterations could utilize competitive growth assays with two or more strains grown in the same well, followed by multiplexed allele-specific RT-qPCR to provide individual measurements that are internally controlled. It could also be used to measure the fitness of strains in outgrowth assays, using samples from animal models or people living with HIV to compare growth rates from diverse viral populations. Viral evolution studies and the selective pressure different drugs exert on the virus could also be evaluated with QuickFit. Finally, the use of genetically defined target cell lines could further reduce variability relative to PBMCs.

## 5. Conclusions

QuickFit is a newly developed method for the quantification of viral growth. This assay is a reproducible, quantitative, and high-throughput approach that is useful for the evaluation of viral fitness. The growth rates determined for several HIV strains by QuickFit were replicable by ELISA but required less hands-on time and exhibited less errors. QuickFit determined and compared the growth rates, carrying capacities, and fitness of different HIV strains and mutants with high precision, and donor-to-donor variability did not significantly impact these values when using cells from the same source. QuickFit also determined the impact on fitness imposed on viruses grown in the presence of ART drugs, highlighting the versatility of this assay and its utility in the field of viral evolution and therapies.

## Data Availability

The raw data supporting the conclusions of this article will be made available by the authors upon request.

## Supplementary Materials

The following supporting information can be downloaded at: https://www.mdpi.com/article/10.3390/v16081320/s1, Supplementary Figure S1. Analysis and modeling of HIV_REJO.c_ *in vitro* replication without data filtering and comparison of ELISA and QuickFit growth curves. Supplementary Figure S2. Determination of HIV_REJO.c_ WT and Pol mutants raw p24 concentration and viral loads and evaluation of growth rates by p24 ELISA. Supplementary Figure S3. Changes in fitness cost for the same isolate are retained despite donor variability. Supplementary Figure S4. Emtricitabine does not impact the RT-qPCR quantification. Supplementary Figure S5. Analysis and modeling of in vitro replication growth curves for four different HIV-1 strains as measured by p24 ELISA and QuickFit RT-qPCR. Supplementary Figure S6. Growth rates for different isolates may vary between donors. Supplementary Table S1. Raw p24 concentration and viral loads. Supplementary Table S2. Individual and average growth rates and carrying capacities determined for HIV_REJO.c_ by p24 ELISA and RT-qPCR with and without data filtering. Supplementary Table S3. Individual and average growth rates and carrying capacities determined for HIV_REJO.c_ WT and Pol mutants by p24 ELISA and RT-qPCR after data filtering. Supplementary Table S4. Individual and average growth rates and carrying capacities determined for different HIV strains by p24 ELISA and RT-qPCR after data filtering. Supplementary Table S5. Individual and average growth rates and carrying capacities determined for different HIV strains by RT-qPCR running a single simulation.
