# Supplementary material for: QuickFit: A High-Throughput RT-qPCR-Based Assay to Quantify Viral Growth and Fitness In Vitro
Source: Viruses. 2024 Aug 19;16(8):1320. doi: 10.3390/v16081320 (PMC11360610; doi:10.3390/v16081320)
Supplement: Supplementary file 1 [file viruses-16-01320-s001.zip › 2024-07-22 - Supplementary Tables.pdf]

Supplementary Table 1. Raw p24 concentration and viral loads.

p24 ELISA Raw pg/mL										
HIV$_{REJO.c}$	Replicate 1					Replicate 2				
Dilution	Day 2	Day 3	Day 4	Day 5	Day 6	Day 2	Day 3	Day 4	Day 5	Day 6
1:2	2.48E+04	5.78E+04	1.43E+05	2.25E+05	2.92E+05	2.42E+04	6.10E+04	1.25E+05	2.22E+05	2.97E+05
1:4	1.60E+04	3.86E+04	9.07E+04	1.60E+05	2.20E+05	1.60E+04	3.73E+04	9.57E+04	1.59E+05	2.11E+05
1:8	1.60E+04	2.72E+04	7.08E+04	1.24E+05	1.88E+05	1.60E+04	2.56E+04	6.96E+04	1.38E+05	1.94E+05
1:16	1.60E+04	1.83E+04	5.31E+04	1.01E+05	1.36E+05	1.60E+04	1.84E+04	5.71E+04	1.24E+05	1.87E+05
1:32	1.60E+04	1.60E+04	3.30E+04	6.70E+04	9.91E+04	1.60E+04	1.60E+04	3.49E+04	7.85E+04	1.23E+05
1:64	1.60E+04	1.60E+04	2.96E+04	6.57E+04	1.06E+05	1.60E+04	1.60E+04	2.17E+04	5.64E+04	1.02E+05
1:128	1.60E+04	1.60E+04	1.60E+04	4.28E+04	7.35E+04	1.60E+04	1.60E+04	1.60E+04	3.99E+04	6.69E+04
1:256	1.60E+04	1.60E+04	1.60E+04	1.60E+04	2.23E+04	1.60E+04	1.60E+04	1.60E+04	2.98E+04	5.79E+04

RT-qPCR Raw GC/mL										
HIV$_{REJO.c}$	Replicate 1					Replicate 2				
Dilution	Day 2	Day 3	Day 4	Day 5	Day 6	Day 2	Day 3	Day 4	Day 5	Day 6
1:2	2.28E+07	3.41E+07	3.51E+07	5.14E+07	5.58E+07	2.61E+07	4.27E+07	5.14E+07	4.41E+07	7.07E+07
1:4	1.26E+07	2.49E+07	4.14E+07	4.01E+07	4.44E+07	1.58E+07	2.01E+07	2.98E+07	2.51E+07	4.06E+07
1:8	6.62E+06	1.43E+07	2.35E+07	2.18E+07	2.93E+07	1.16E+07	1.58E+07	1.80E+07	1.91E+07	2.38E+07
1:16	5.60E+06	1.15E+07	1.77E+07	1.47E+07	2.56E+07	7.70E+06	1.12E+07	1.71E+07	1.73E+07	2.23E+07
1:32	2.42E+06	3.25E+06	8.51E+06	7.75E+06	1.51E+07	5.72E+06	7.04E+06	1.60E+07	1.42E+07	2.47E+07
1:64	1.08E+06	1.96E+06	4.01E+06	4.61E+06	7.08E+06	2.21E+06	4.45E+06	1.07E+07	8.07E+06	1.53E+07
1:128	9.31E+05	1.07E+06	2.85E+06	3.91E+06	5.41E+06	1.26E+06	1.24E+06	4.19E+06	4.97E+06	9.04E+06
1:256	4.42E+05	6.66E+05	2.37E+06	2.52E+06	3.31E+06	4.50E+05	6.39E+05	1.89E+06	1.79E+06	3.46E+06

†Greyed-out rows represent sample sets excluded from downstream analyses according to inclusion and exclusion criteria.

Supplementary Table 2. Individual and average growth rates and carrying capacities determined for $HIV_{REJO.c}$ by p24 ELISA and RT-qPCR with and without data filtering.

$HIV_{REJO.c}$	p24 ELISA		Unfiltered RT-qPCR Data		Filtered RT-qPCR Data	
**K (pg/mL or GC/mL)***	5.96E+05		3.16E+07		3.85E+07	
**Dilution**	**Replicate 1**	**Replicate 2**	**Replicate 1**	**Replicate 2**	**Replicate 1**	**Replicate 2**
**1:2**	0.99	0.99	0.88	0.95		
**1:4**	0.99	0.99	1.06	0.75		
**1:8**	0.88	0.89	0.92	0.62	0.81	
**1:16**	0.77	0.85	0.83	0.69	0.76	
**1:32**	0.64	0.69	0.76	0.80	0.74	0.75
**1:64**	0.64	0.61	0.73	0.92	0.72	0.82
**1:128**			0.67	0.72	0.69	0.71
**1:256**			0.80	0.71	0.77	0.71
**Average r ± SD****	**0.83 ± 0.14**		**0.80 ± 0.12**		**0.75 ± 0.04**	

***Carrying capacity values (K) correspond to the average highest value Viral Growth indicator (either p24 pg/mL or GC/mL) that could be sustained in a steady state.**

****The r value represents the replication rate measured for each data set.**

†Greyed-out rows represent sample sets excluded from downstream analyses according to inclusion and exclusion criteria.

Supplementary Table 3. Individual and average growth rates and carrying capacities determined for HIV$_{REJO.c}$ WT and Pol mutants by p24 ELISA and RT-qPCR after data filtering.

HIV$_{REJO.c}$	ELISA		Filtered qPCR Data		Pol M184I	ELISA		Filtered qPCR Data		Pol M184V	ELISA		Filtered qPCR Data	
K (pg/mL or GC/mL)*	8.00E+04		1.70E+07		K (pg/mL or GC/mL)*	8.00E+04		1.70E+07		K (pg/mL or GC/mL)*	8.00E+04		1.70E+07	
Dilution	Replicate 1	Replicate 2	Replicate 1	Replicate 2	Dilution	Replicate 1	Replicate 2	Replicate 1	Replicate 2	Dilution	Replicate 1	Replicate 2	Replicate 1	Replicate 2
1:2	0.93	0.77			1:2	0.71				1:2	0.71	0.73		
1:4	0.77	0.70			1:4	0.55	0.53			1:4	0.63	0.68		
1:8	0.59				1:8					1:8		0.58		0.59
1:16			1.01	0.79	1:16					1:16		0.48	0.56	0.47
1:32			0.88	0.86	1:32			0.62	0.64	1:32			0.59	0.48
1:64			0.81	0.92	1:64			0.64	0.60	1:64			0.57	0.61
1:128			0.73	0.81	1:128			0.66	0.71	1:128			0.55	0.51
1:256					1:256				0.70	1:256				
Average r ± SD**	0.75 ± 0.12		0.85 ± 0.09		Average r ± SD	0.59 ± 0.1		0.65 ± 0.04		Average r ± SD	0.64 ± 0.09		0.55 ± 0.05	
HIV$_{REJO.c}$ + FTC	ELISA		Filtered qPCR Data		Pol M184I + FTC	ELISA		Filtered qPCR Data		Pol M184V + FTC	ELISA		Filtered qPCR Data	
K (pg/mL or GC/mL)*	8.00E+04		1.70E+07		K (pg/mL or GC/mL)*	8.00E+04		1.70E+07		K (pg/mL or GC/mL)*	8.00E+04		1.70E+07	
Dilution	Replicate 1	Replicate 2	Replicate 1	Replicate 2	Dilution	Replicate 1	Replicate 2	Replicate 1	Replicate 2	Dilution	Replicate 1	Replicate 2	Replicate 1	Replicate 2
1:2					1:2	0.69	0.58			1:2	0.55	0.49		
1:4					1:4	0.58	0.47			1:4				
1:8					1:8					1:8				0.51
1:16					1:16					1:16			0.54	0.51
1:32					1:32			0.35	0.38	1:32			0.43	0.45
1:64					1:64			0.42		1:64			0.49	0.50
1:128					1:128					1:128				
1:256					1:256					1:256				
Average r ± SD	NA		NA		Average r ± SD	0.58 ± 0.09		0.38 ± 0.03		Average r ± SD	0.52 ± 0.04		0.49 ± 0.04	

***Carrying capacity values (K) correspond to the average highest value Viral Growth indicator (either p24 pg/mL or GC/mL) that could be sustained in a steady state.**
****The r value represents the replication rate measured for each data set.**
†Greyed-out rows represent sample sets excluded from downstream analyses according to inclusion and exclusion criteria.

Supplementary Table 4. Individual and average growth rates and carrying capacities determined for different HIV strains by p24 ELISA and RT-qPCR after data filtering.

HIV_{NL4-3}	ELISA		Filtered qPCR Data		HIV_{JR-CSF}	ELISA		Filtered qPCR Data	
**K (pg/mL or GC/mL)***	4.09E+05		4.53E+11		**K (pg/mL or GC/mL)***	2.97E+05		1.14E+08	
**Dilution**	**Replicate 1**	**Replicate 2**	**Replicate 1**	**Replicate 2**	**Dilution**	**Replicate 1**	**Replicate 2**	**Replicate 1**	**Replicate 2**
**1:2**	0.87	0.75			**1:2**				
**1:4**	0.92	0.87			**1:4**			0.82	0.96
**1:8**	0.94	0.92	0.59	0.56	**1:8**	1.13	1.51	0.94	1.13
**1:16**	0.88	0.83	0.74	0.72	**1:16**	0.86	1.02	1.20	1.27
**1:32**	0.67	0.76	0.98	0.87	**1:32**	0.71	0.71	1.35	1.50
**1:64**	0.66	0.63			**1:64**				
**1:128**					**1:128**				
**1:256**					**1:256**				
**Average r ± SD**	**0.81 ± 0.11**		**0.74 ± 0.16**		**Average r ± SD**	**0.99 ± 0.3**		**1.15 ± 0.23**	
$HIV_{89.6}$	**ELISA**		**Filtered qPCR Data**		HIV_{BF520}	**ELISA**		**Filtered qPCR Data**	
**K (pg/mL or GC/mL)***	2.46E+05		3.52E+08		**K (pg/mL or GC/mL)***	5.40E+05		7.00E+09	
**Dilution**	**Replicate 1**	**Replicate 2**	**Replicate 1**	**Replicate 2**	**Dilution**	**Replicate 1**	**Replicate 2**	**Replicate 1**	**Replicate 2**
**1:2**	0.88	1.19			**1:2**	1.35	1.00		
**1:4**	0.74	1.07	0.97	0.91	**1:4**				
**1:8**	0.68	1.11	1.04	0.90	**1:8**			0.92	0.84
**1:16**		1.05	1.16	1.10	**1:16**			0.85	0.87
**1:32**		0.97	1.13	1.04	**1:32**			1.02	1.12
**1:64**		0.83	1.23		**1:64**				
**1:128**		0.92			**1:128**				
**1:256**					**1:256**				
**Average r ± SD**	**0.94 ± 0.16**		**1.05 ± 0.11**		**Average r ± SD**	**1.18 ± 0.25**		**0.94 ± 0.11**	

[*]Carrying capacity values (K) correspond to the average highest value Viral Growth indicator (either p24 pg/mL or GC/mL) that could be sustained in a steady state.
[**]The r value represents the replication rate measured for each data set.
[†]Greyed-out rows represent sample sets excluded from downstream analyses according to inclusion and exclusion criteria.

Supplementary Table 5. Individual and average growth rates and carrying capacities determined for different HIV strains by RT-qPCR running a single simulation.

HIV Strain	$HIV_{REJO.c}$		HIV_{NL4-3}		HIV_{JR-CSF}		$HIV_{89.6}$		HIV_{BF520}	
K (GC/mL)*	2.34E+07		9.61E+09		8.73E+07		3.97E+08		9.61E+09	
Dilution	Replicate 1	Replicate 2	Replicate 1	Replicate 2	Replicate 1	Replicate 2	Replicate 1	Replicate 2	Replicate 1	Replicate 2
1:2										
1:4					0.93	1.03	0.95	0.88		
1:8	0.82		0.68	0.66	1.02	1.18	1.03	0.86	0.86	0.79
1:16	0.79		0.80	0.78	1.24	1.27	1.16	1.10	0.81	0.84
1:32	0.78	0.72	1.02	0.91	1.36	1.49	1.12	1.03	1.02	1.09
1:64	0.75	0.83					1.23			
1:128	0.69	0.74								
1:256	0.78	0.72								
Average r ± SD**	**0.76 ± 0.05**		**0.81 ± 0.14**		**1.19 ± 0.19**		**0.90 ± 0.12**		**1.04 ± 0.13**	
Doubling Time⁺	**31.50**		**29.77**		**20.15**		**23.10**		**26.67**	

*Carrying capacity values correspond to the average highest value of GC/mL that could be sustained in a steady state for each isolate.

**The r value represents the replication rate measure for each data set.

⁺The Doubling Time represents the number of hours it takes each isolate to double.

†Greyed-out rows represent sample sets excluded from downstream analyses according to inclusion and exclusion criteria.
