# Supplementary material for: QuickFit: A High-Throughput RT-qPCR-Based Assay to Quantify Viral Growth and Fitness In Vitro
Source: Viruses. 2024 Aug 19;16(8):1320. doi: 10.3390/v16081320 (PMC11360610; doi:10.3390/v16081320)
Supplement: Supplementary file 1 [file viruses-16-01320-s001.zip › 2024-07-22 - Supp Figures.pdf]

SUPPLEMENTARY FIGURES

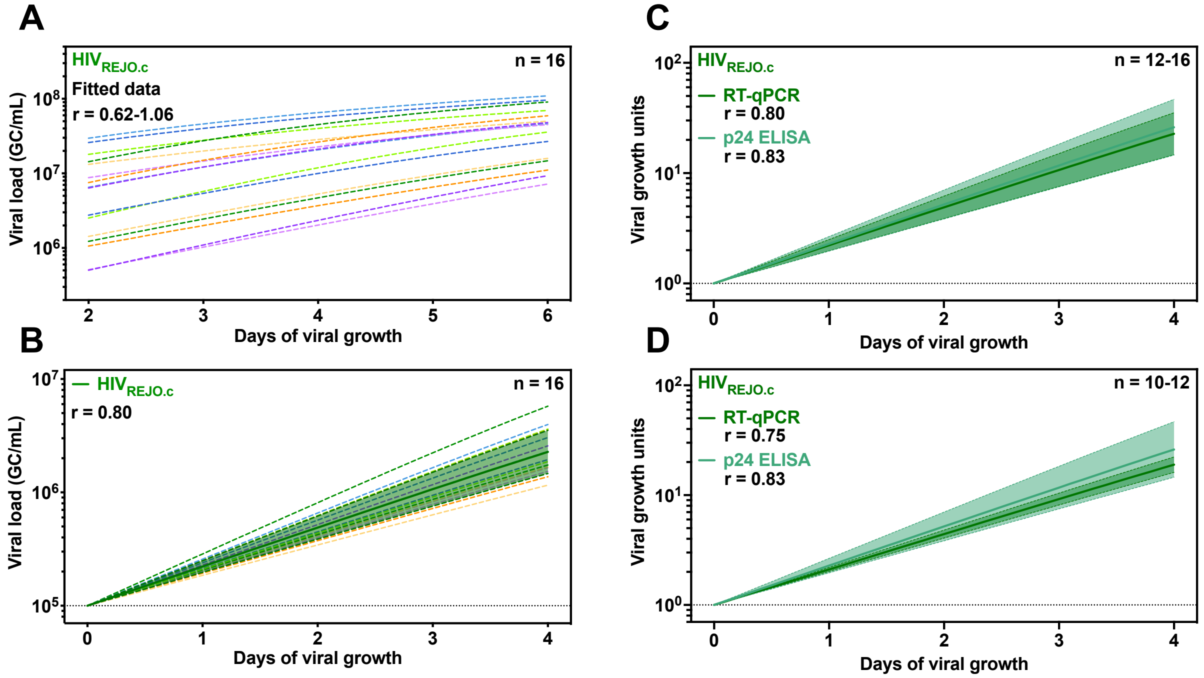

Supplementary Figure 1. Analysis and modeling of $HIV_{REJO.c}$ *in vitro* replication without data filtering and comparison of ELISA and QuickFit growth curves. (**A**) Viral loads (GC/mL) were determined for $HIV_{REJO.c}$ by QuickFit RT-qPCR and all the data from each individual well were used to determine growth rates and carrying capacities by running an NMLE modeling with a half-maximal equation in the MonolixSuite software. A total of 16 samples at 5 different time points were evaluated, and matching light-dark colors represent duplicates. (**B**) Viral growth (GC/mL) was calculated by normalizing the initial value on day 0 to $1x10^5$ GC/mL and subsequently interpolating the remaining values from days 1 to 4 based on the fit curve model. Overlaid is the mean growth rate and the 95% confidence interval. (**C**) Viral growth (p24 concentration or GC/mL) of inclusion and exclusion criteria filtered data was calculated by normalizing the initial value on day 0 to either 1.00 pg/mL or 1.00 GC/mL and subsequently interpolating the remaining values from days 1 to 4 based on the fit curve model. Overlaid is the mean growth rate and the 95% confidence interval. The r value represents the average growth rate determined for all included samples by each assay.

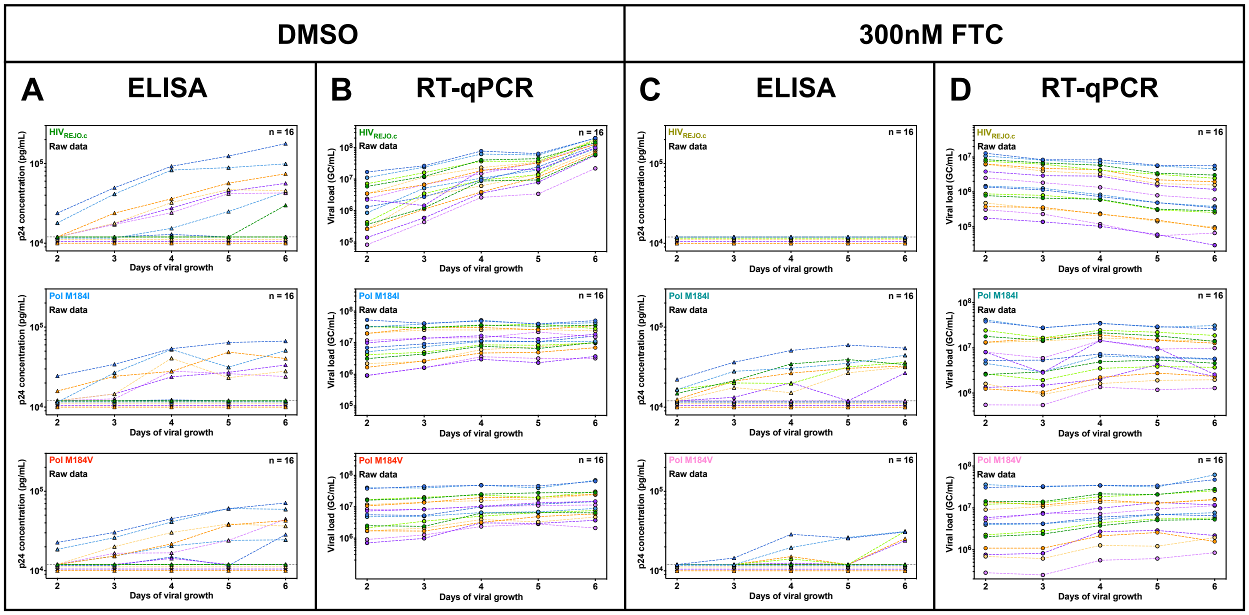

E

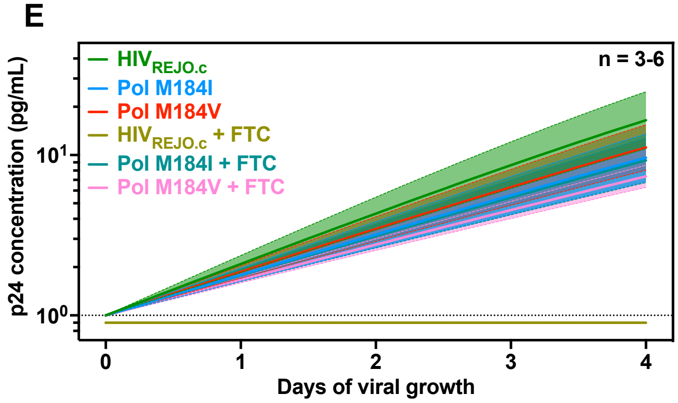

Mutation tested	r ± SD (n)	Fitness cost
$HIV_{REJO.c}$	0.75 ± 0.12 (5)	1.00
Pol M184I	0.59 ± 0.1 (3)	1.27
Pol M184V	0.64 ± 0.09 (6)	1.18
$HIV_{REJO.c}$ + FTC	NA	NA
Pol M184I + FTC	0.58 ± 0.09 (4)	1.29
Pol M184V + FTC	0.52 ± 0.04 (2)	1.45

Supplementary Figure 2. Determination of $HIV_{REJO.c}$ WT and Pol mutants raw p24 concentration and viral loads and evaluation of growth rates by p24 ELISA. Single-point mutations for the pol gene were introduced in the $HIV_{REJO.c}$ IMC backbone and p24 ELISA and RT-qPCR were performed with samples obtained from the QuickFit pipeline experimental setup. These strains were also grown in the presence of 300nM emtricitabine (FTC) or DMSO as a vehicle control. Determined raw p24 concentrations (pg/mL) for the different strains grown in (**A**) DMSO- or (**C**) FTC - containing media and raw viral loads (GC/mL) for the different strains grown in (**B**) DMSO- or (**D**) FTC - containing media are shown. A total of 16 samples at 5 different time points were evaluated. The limits of detection (LOD) shown correspond to the standard curve working range after sample dilution correction. (**E**) Viral growth (p24 concentration) was calculated by normalizing the initial value of each sample (after inclusion and exclusion criteria filtering) on day 0 to 1.00 pg/mL and subsequently interpolating the remaining values from days 1 to 4 based on the fit curve model. Overlaid is the mean growth rate and the 95% confidence interval. The table to the right shows the r value (growth rates) ± the standard deviation for each strain tested for both conditions. The sample size (n) references the number of wells included in the analysis after filtering with inclusion and exclusion criteria. The fitness cost represents the doubling rate of each strain relative to the WT $HIV_{REJO.c}$ without FTC. **NA**: No growth was detected.

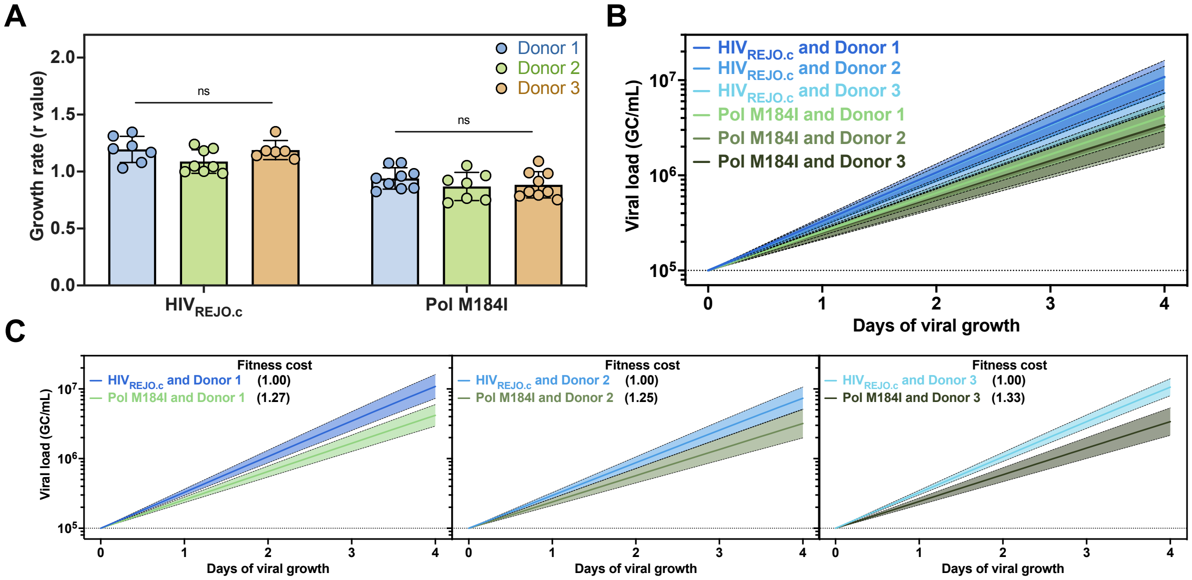

Supplementary Figure 3. Changes in fitness cost for the same isolate are retained despite donor variability. The growth rates for the $HIV_{REJO.c}$ WT and Pol M184I mutant strains were evaluated using different PBMC donors and constraining the simulations to a shared K value. (**A**) Comparison of r values determined for both strains for each donor (ns: non-significant; Two-way ANOVA with Šídák *post hoc* analysis). (**B**) Normalized growth curves were generated for both strains and for each donor independently. (**C**) Pairwise comparison of the normalized growth curves generated for both strains and for each donor. The fitness cost represents the doubling rate of each strain relative to the WT $HIV_{REJO.c}$.

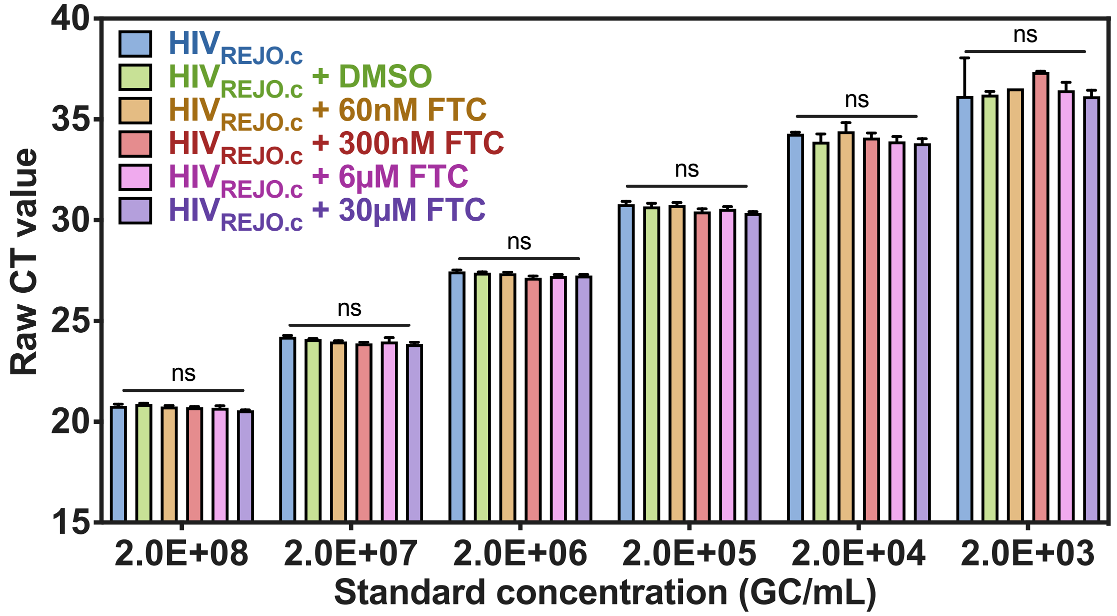

Supplementary Figure 4. Emtricitabine does not impact the RT-qPCR quantification. Serial dilutions of a previously tittered stock of $HIV_{REJO.c}$ were quantified by RT-qPCR in the presence of increasing concentrations of emtricitabine (FTC, an RT inhibitor) ranging from 35 ng/mL (60nM) to 700 ng/mL (30µM). Raw CT values measured are shown. The highest FTC concentration tested here is 10 times higher than the one tested in the experiments shown in **Figure 3**. No statistically significant differences were found for any of the different FTC concentrations at any specific standard concentrations (Two-way ANOVA with Šídák *post hoc* analysis).

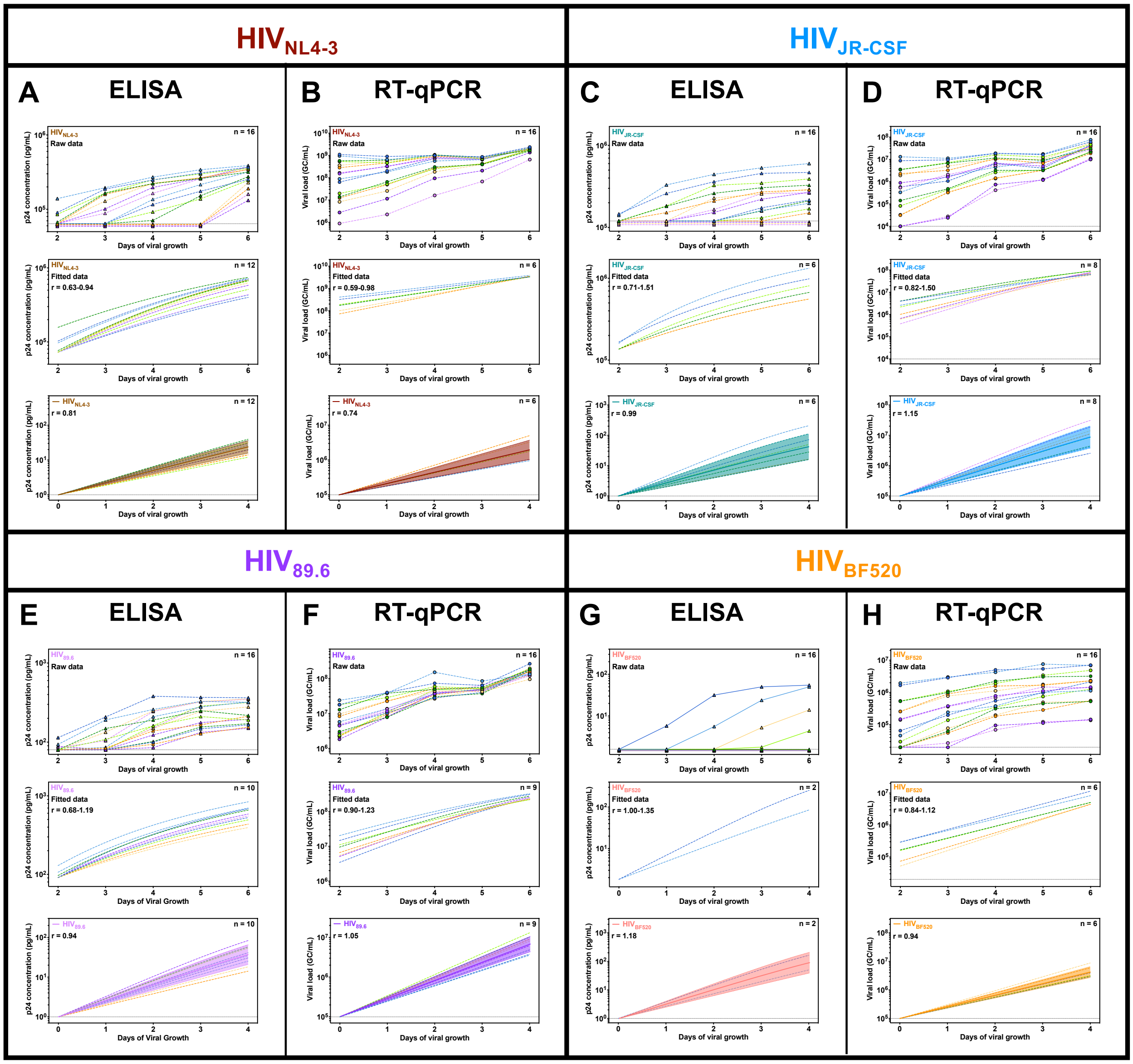

Supplementary Figure 5. Analysis and modeling of in vitro replication growth curves for four different HIV-1 strains as measured by p24 ELISA and QuickFit RT-qPCR. Both p24 ELISA (**left**) and QuickFit RT-qPCR (**right**) were performed for the four different HIV-1 strains (**A,B**) $HIV_{NL4\text{-}3}$, (**C,D**) $HIV_{JR\text{-}CSF}$, (**E,F**) $HIV_{89.6}$, and (**G,H**) HIV_{BF520}. (**A,C,E,G, top**) Determined raw p24 concentrations (pg/mL) and (**B,D,F,H, top**) viral loads (GC/mL) are shown. A total of 16 samples at 5 different time points were evaluated. The limits of detection (LOD) shown correspond to the standard curve working range after sample dilution correction. (**Middle**) Samples were filtered using inclusion and exclusion criteria, and the remaining data were used to determine growth rates and carrying capacities by running an NMLE modeling with a half-maximal equation in the MonolixSuite software. (**Bottom**) Normalized viral growth (p24 concentration or GC/mL) was calculated by normalizing the initial value on day 0 to 1.00 pg/mL or $1x10^5$ GC/mL and subsequently interpolating the remaining values from days 1 to 4 based on the fit curve model. Overlaid is the mean growth rate and the 95% confidence interval. The r

value represents the average growth rate determined for all included samples by each assay.

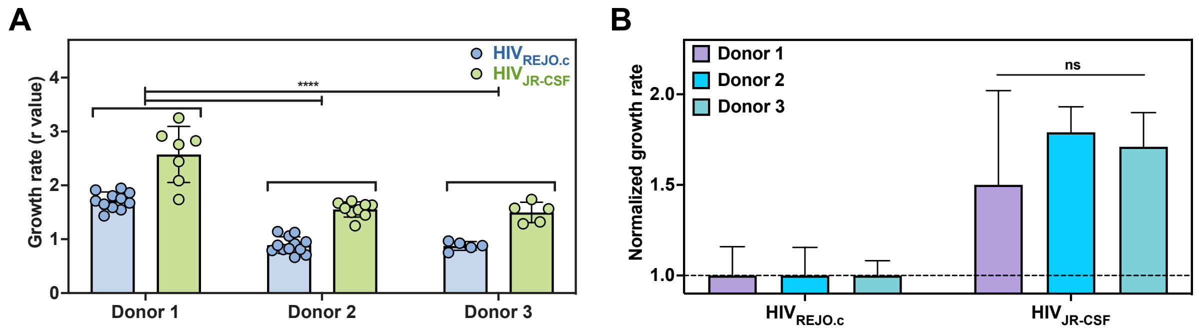

Supplementary Figure 6. Growth rates for different isolates may vary between donors. The growth rates for $HIV_{REJO.c}$ and $HIV_{JR\text{-}CSF}$ were determined using PBMCs from three different donors. A single simulation, including all strains and all donors, was performed without constraining the carrying capacity to a shared value. (**A**) Comparison of growth rates between each donor (******:** p<0.001; Two-way ANOVA with Šídák *post hoc* analysis). (**B**) The growth rate of $HIV_{JR\text{-}CSF}$ for each donor was normalized to that of the corresponding $HIV_{REJO.}$c and then compared between each other to determine whether the doubling rate ratio was maintained across donors (ns: non-significant; Two-way ANOVA with Šídák *post hoc* analysis).
